# No Change? A Grounded Theory Analysis of Depressed Patients' Perspectives on Non-improvement in Psychotherapy

**DOI:** 10.3389/fpsyg.2019.00588

**Published:** 2019-03-26

**Authors:** Melissa Miléna De Smet, Reitske Meganck, Kimberly Van Nieuwenhove, Femke L. Truijens, Mattias Desmet

**Affiliations:** ^1^Department of Psychoanalysis and Clinical Consulting, Ghent University, Ghent, Belgium; ^2^Fonds Wetenschappelijk Onderzoek, Brussels, Belgium

**Keywords:** non-improvement, psychotherapy, outcome research, grounded theory, depression-psychology, mixed-method analyses, qualitative and quantitative methods, patient perspective

## Abstract

**Aim:** Understanding the effects of psychotherapy is a crucial concern for both research and clinical practice, especially when outcome tends to be negative. Yet, while outcome is predominantly evaluated by means of quantitative pre-post outcome questionnaires, it remains unclear what this actually means for patients in their daily lives. To explore this meaning, it is imperative to combine treatment evaluation with quantitative and qualitative outcome measures. This study investigates the phenomenon of non-improvement in psychotherapy, by complementing quantitative pre-post outcome scores that indicate no reliable change in depression symptoms with a qualitative inquiry of patients' perspectives.

**Methods:** The study took place in the context of a Randomised Controlled Trial evaluating time-limited psychodynamic and cognitive behavioral therapy for major depression. A mixed methods study was conducted including patients' pre-post outcome scores on the BDI-II-NL and post treatment Client Change Interviews. Nineteen patients whose data showed no reliable change in depression symptoms were selected. A grounded theory analysis was conducted on the transcripts of patients' interviews.

**Findings:** From the patients' perspective, non-improvement can be understood as being stuck between knowing versus doing, resulting in a stalemate. Positive changes (mental stability, personal strength, and insight) were stimulated by therapy offering moments of self-reflection and guidance, the benevolent therapist approach and the context as important motivations. Remaining issues (ambition to change but inability to do so) were attributed to the therapy hitting its limits, patients' resistance and impossibility and the context as a source of distress. “No change” in outcome scores therefore seems to involve a “partial change” when considering the patients' perspectives.

**Conclusion:** The study shows the value of integrating qualitative first-person analyses into standard quantitative outcome evaluation and particularly for understanding the phenomenon of non-improvement. It argues for more multi-method and multi-perspective research to gain a better understanding of (negative) outcome and treatment effects. Implications for both research and practice are discussed.

Negative outcome or nonresponse to treatment is undeniably part of clinical practice. It is estimated that 5 to 10% of patients deteriorates in therapy (Cooper, 2008; Lambert, 2013), and a proportion of 35 to 40% of the participants in clinical trials do not improve (Lambert, 2007). A better understanding of negative outcome and treatment effects is crucial for both research and clinical practice, yet outcome research has focused predominantly on capturing positive change and “what works,” while less is known about non-improvement or what it actually means when treatments fail (Barlow, 2010).

There is no uniform understanding of negative outcome, nor is there agreement on the definition of treatment failure (Lambert, 2011; Lampropoulos, 2011). “Negative outcome” and “negative therapeutic effects” are often used as synonyms, although they do not have a one-on-one relationship, as negative outcome is not necessarily caused by therapy (Mays and Franks, 1985; Mohr, 1995). Depending on the perspective (e.g., patient, therapist, researcher), the type of outcome (e.g., symptoms, quality of life), measurement method (e.g., quantitative or qualitative) and time point (e.g., post treatment or follow-up) being used for treatment evaluation, the conception of outcome and treatment effects varies (Lampropoulos, 2011).

In outcome research, outcome and treatment effects are typically evaluated using statistical tests of significance that provide an indication of the reliability of the measured change. Statistical significance shows that an outcome difference is larger than could have been expected by mere chance. Clinical significance shows whether such a statistical effect is also clinically meaningful (i.e., change toward a normal level of functioning) (Jacobson et al., 1999; Ogles et al., 2001; Lambert et al., 2008; Lambert and Ogles, 2009). Based on the Jacobson and Truax widely used method for clinical significance, outcome can be classified into four categories: (1) recovery (i.e., clinically significant change), (2) improvement (i.e., reliable change), (3) no reliable change and (4) deterioration (i.e., reliable change in the negative direction). Generally, the first category “recovery” is taken as the gold standard outcome and treatment goal: a reliable decrease in symptoms^1^ and return to a non-clinical level of functioning. When neither criterion is met, it is concluded that patients remained “unchanged” in comparison to their level of functioning prior to treatment (Jacobson and Truax, 1991).

Despite the added value of clinical significance testing of measured changes, this type of statistical outcome classification cannot overcome the limitations that are voices for standard outcome research (Hill et al., 2013). Quantitative pre-post outcome evaluation is criticised for relying predominantly on one-dimensional rating scales, most often symptom-based (Braakmann, 2015), and consequently, for offering only an incomplete approximation of the multi-dimensional nature of human functioning (Kazdin, 2001; Hill et al., 2013). The possible discrepancy between what is measured with outcome questionnaires and what is meaningful in patients' daily life has been problematised: a patient's outcome score might fall within the non-clinical range while it does not reflect the person's functioning (Kazdin, 2011). Real-life contextualisation is necessary in order to make sense of what changes in scores (or the lack thereof) actually mean for an individual (Blanton and Jaccard, 2006; Kazdin, 2006). The latter is typically missing in large sample standardised outcome studies, and consequently, the dissemination of research findings into clinical practice generally fails (Kazdin, 2008).

The past decades have seen an accumulation of qualitative studies attempting to contribute to overcoming this research-practice barrier, gradually offering a more central role to the voice of patients (Levitt et al., 2016). Qualitative research focusing on patients' experiences of outcome has provided a diverse picture of treatment-related changes (McLeod, 2011). Apart from symptomatic changes, alterations on the level of patients' self, life, interpersonal relations, and self-understanding have been observed (e.g., Binder et al., 2009). The largest strand of qualitative psychotherapy research has focused on patients' experiences of therapy, aiming to identify helping and hindering aspects (McLeod, 2013). Hindering elements in therapy that have been mentioned by patients are contra-productive therapist features (e.g., being unsure, absent or non-responsive, lack of direction and advise in therapy), patients' own difficulties to express or get in touch with their feelings and lack of commitment and motivation, and a lack of trust between patient and therapist (Paulson et al., 2001; von Below and Werbart, 2012) and so forth. On the other hand, a joint exploration of difficulties and experiencing warmth, understanding and empathy in the relationship with the therapist were found to be helpful for patients (Timulak and Lietaer, 2001; Lilliengren and Werbart, 2005; Bohart and Wade, 2013).

Interestingly, findings from qualitative outcome studies shine a somewhat more pessimistic light on psychotherapy outcome than is typically observed in quantitative studies. In general, patients tend to be more critical about therapy during interviews, for instance, expressing disappointment about unaltered core problems or ambivalence about the gains of therapy (McLeod, 2013). Moreover, research findings suggest that, patients' treatment satisfaction does not correspond to changes in outcome scores. Werbart et al. (2015), for instance, observed that only three out of twenty patients with a nonimproved or deteriorated outcome also clearly indicated to be dissatisfied about treatment.

Nonetheless, the association between quantitative and qualitative evaluations of therapy and outcome remains unclear (Timulak and Creaner, 2010). Mixed-methods studies have amassed in the past couple of years, though whether and how patients' experiences correspond to quantitative outcome evaluation is underexplored (McLeod, 2013). The few studies that have been executed differ in the extent to which qualitative and quantitative findings show an accord (see Svanborg et al., 2008 vs. Klein and Elliott, 2006). The study of McElvaney and Timulak (2013) found only little differences between patients classified as “recovered/ improved” and “unchanged/ deteriorated” regarding their experience of therapy. As the strict demarcation of “poor” and “good” outcome does not appear in qualitative inquiry, questions can be raised about how representative such a statistical distinction is for the clinical meaning of outcome for individual patients (see Lambert and Ogles, 2009).

So far, the meaning of negative or poor outcome—distinguished by means of standard outcome measures—in relation to patients' subjective experiences, remains underexplored. As non-improvement and worsening are likely distinct phenomena with potential different clinical implications (Mohr, 1995; Lambert, 2011), more focused investigations are required in order to grasp the phenomenon of non-improvement (in contrast to the approach of McElvaney and Timulak, 2013, who studied unchanged and deteriorated cases together). In past endeavours, most of the studies have focused on deterioration or other “extreme cases,” yet little attention has been allotted to understanding treatment nonresponse or patient non-improvement specifically (Lambert, 2011). Given the observation that lack of improvement occurs in a significant number of cases, and considerably more frequently than deterioration, this lack of attention is striking (Lambert, 2007, 2013). Importantly, gaining a better understanding of cases who seemingly have not moved forward or backward, will contribute to a more thorough and nuanced understanding of treatment-response and outcome in general. More specifically, this nuanced understanding is pivotal to elaborate the clinical meaning of outcome for patients themselves.

The integration of multiple methods and specifically the comparison of quantitative and qualitative methods is an indispensable development for the field of psychotherapy research (McLeod, 2013; Bowie et al., 2016). The current study therefore provides a mixed-method analysis of patients suffering from major depression. Major depression is one of the most prevalent mental disorders worldwide (WHO, 2017), and previous research has shown symptomatic evaluation of change alone cannot live up to the task of representing depressed patients' experience of outcome (Zimmerman et al., 2006, 2012). Based on this representative case, the present study aims to complement quantitative pre-post outcome scores indicating no reliable change in depression symptoms with a qualitative inquiry of depressed patients' perspective. In doing so, we move beyond the level of description (i.e., a lack of change in symptom scores) and toward a level of in-depth understanding (i.e., patients' subjective experience). Finally, instead of adopting a single focus on experiences of outcome or experiences of therapy, the present study aims to understand their interrelation as well as the broader context of potential influences, as these are typically not limited to therapeutic features alone (Drisko, 2004; De Smet and Meganck, 2018).

The current study investigates how non-improvement in pre-to-post symptom severity can be understood in relation the experience of depressed patients themselves. We examine: (1) which potential changes patients have experienced and which factors can help to explain these changes from their perspective; (2) which potential issues remained and which factors can help explain these remaining issues according to patients; (3) how patients' perspective on non-improvement relates to the quantitative outcome evaluation of non-improvement (or no reliable change) in symptom severity. For the purpose of the study, the term “non-improvement” is used to indicate a specific definition of negative or poor outcome in accordance to the widely used statistical concept of a lack of reliable change in outcome scores (cf. Jacobson and Truax, 1991). We use this categorisation as a starting point to be able to *broaden* this influential framework of understanding, by nuancing it based on patients' perspectives.

## Methodology

An explanatory sequential mixed-methods study was conducted, comprising a quantitative pre-post outcome evaluation as well as a qualitative analysis of nonimproved patients' perspective. The study is “explanatory” as the focus is on understanding non-improvement in-depth, and “sequential” because, even though quantitative and qualitative data were gathered simultaneously, both strands were analysed independently and integrated at the phase of interpretation. The design can be summarised as “quan →QUAL”: The qualitative analyses build on the quantitative outcome evaluation yet becoming the most important focus of the explanatory study; “the quantitative study (quan) is in service of the more dominant qualitative (QUAL) one” (Hesse-Biber, 2010, p.71). In the current study, a first phase comprised a quantitative outcome evaluation, based on which the target sample was selected. In a second phase, the corresponding interviews were qualitatively analysed. Integration and comparison of the two strands allowed for a better understanding of both the quantitative and qualitative outcome findings. Given the aim for in-depth exploration of patients' experienced changes, as well as understanding of the processes and factors that may explain those experienced changes, a grounded theory approach was selected as method of choice for the qualitative analyses (Strauss and Corbin, 1990). Grounded theory can be used to provide description and interpretation, with the aim to generate conceptual models that can consecutively be translated into further hypotheses (Fassinger, 2005; Charmaz, 2014). For our purposes, thus, this method seemed well-suited to build a thorough understanding of negative outcome and non-improvement from patients' perspective.

### Setting

This study is based on data from the Ghent Psychotherapy Study (GPS), an RCT on the treatment of major depression; the trial has been registered on Open Science Framework (ISRCTN 17130982). For a specific description of the GPS context and methodology, we refer to the pre-registered study protocol (Meganck et al., 2017). Patients in this study were recruited via social media and general practitioners in the area of Ghent, Belgium. Patients included in the study qualified for a diagnosis of Major Depressive Disorder, measured by the Rating Scale for Depression (Hamilton, 1967) and Structured Clinical Interview for DSM-IV-TR (First et al., 2002), both well-established and frequently used interview-based instruments in depression studies (Nezu et al., 2000). The assessment interviews were conducted by six postgraduate research assistants trained in the respective procedures. Further eligibility criteria were sufficient knowledge of the Dutch language and age between 18 and 65; patients with a primary diagnosis of substance abuse, acute psychosis and suicidal ideations were excluded. Patients were randomly assigned to time-limited Cognitive Behavioral Therapy (CBT) or Psychodynamic Therapy (PDT). Patients progress was evaluated using questionnaires accompanying every session, interviews were conducted prior to treatment, around the eighth session and after treatment termination. The follow-up period of the study spans 2 years (ongoing) and consists of 4 interviews and quantitative assessment. This study was approved by the Ethical Committee of the University Hospital of Ghent University (Belgium; EC/2015/0085). All participants gave written informed consent in accordance with the Declaration of Helsinki.

### Treatment

Treatment consisted of CBT and PDT for major depression, two types of therapy that can be distinguished based on their directive (i.e., CBT) and exploration (i.e., PDT) style of interventions. Therapy was provided by one of four therapists in each approach. Both treatments were manualised and time-limited, consisting of 16–20 sessions. Treatment was delivered with an average frequency of one session per week; sessions lasted approximately 45 min. The CBT manual was based on the Cognitive-Behavioral Protocol for Depression by Bockting and Huibers (2011). The PDT manual was based on the Supportive-Expressive Time Limited manual for Major Depressive Disorder by Luborsky (1984) and Leichsenring and Schauenburg (2014). Therapists had an average age of 33 (*SD* = 9.6) and had 3 to 8 years of relevant clinical experience and training in CBT or PDT. In the study, all therapists received 2 days of training, one patient to practice the treatment manual and the research procedure under supervision, and bi-weekly supervision sessions throughout the study.

### Instruments

#### Beck Depression Inventory

The Beck Depression Inventory (BDI-II-NL; Beck et al., 1996; van der Does, 2002)^2^ is a measure of self-reported depression severity. The questionnaire consists of 21 items that are scored on a scale of 0 to 3 and is divided into a cognitive, somatic and affective subscale. A total score between 0 and 13 indicates minimal depression, 14–19 mild depression, 20–28 moderate depression, 29–63 severe depression. The questionnaire shows good validity and reliability (van der Does, 2002).

#### Semi-structured Interview

An adjusted version of the semi-structured Client Change Interview (CCI; Elliott et al., 2001) was administered. The interview guide was constructed to evoke participants' experiences of therapy, the changes they believe occurred during therapy, and what they believe influenced these changes, for instance, helping and hindering aspects of therapy. Every interview started with the open questions: “How are you doing in general?” and “How are you feeling compared to when you started therapy?” Subsequently, patients were asked more specifically about experienced changes: “Which changes have you noticed since the start of therapy (e.g., in relation to others, at school/work, in your emotional wellbeing)?” and the role of therapy or other factors: “How did therapy contribute to these changes?” and “What other factors (outside of therapy) do you think have contributed to these changes?” Patients were also explicitly asked about negative changes or lack of change: “Is there something that did not change or that you would like to change in the future?”; “Did something change in a negative sense during therapy?” All interviews were conducted at the psychology department (Ghent University, Belgium) in the week following therapy termination. Interviews lasted 60 min on average. Interviews were audiotaped, and transcripts were analysed using Nvivo 11 (QSR International).

### Quantitative Outcome Classification on the BDI-II-NL

**Figure 1:**
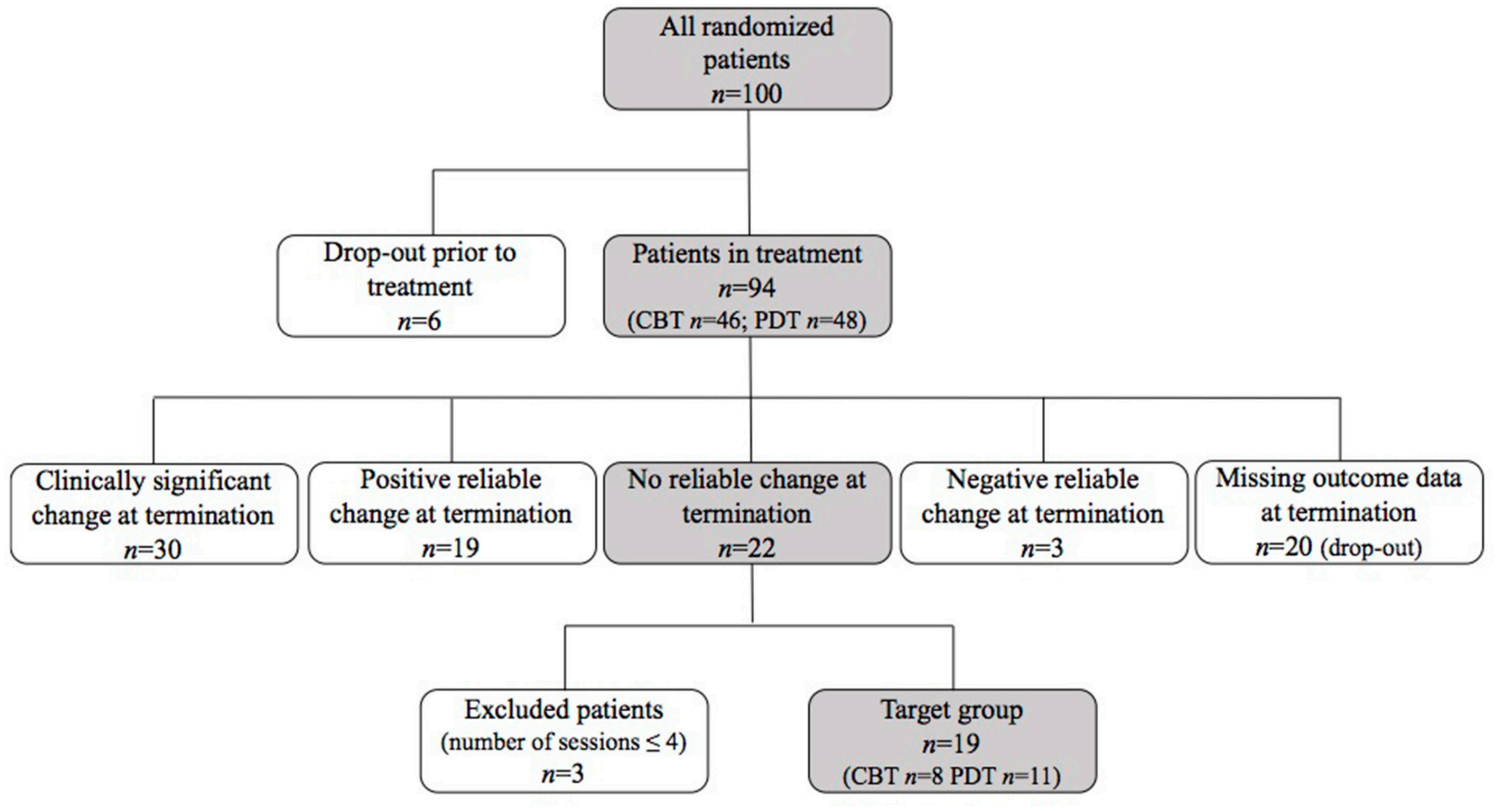
Flow chart of sample selection.

Participants were classified in terms of reliable change and clinically significant change based on the Jacobson and Truax (1991) method for outcome classification. Patients self-reported symptom severity was measured prior to therapy and 1 week after treatment ended. The outcome scores of the patient population were compared to Dutch norms (van der Does, 2002). In order to reach reliable change for the BDI-II-NL total score, a person must show a decrease in scores equal to or larger than 9.6. The cut-off between the clinical and nonclinical population for the Dutch BDI is set at 11.3 (based on the internal consistency of 0.92; van der Does, 2002). This leads to four possible outcomes: Clinically significant change (CS; a decrease in scores equal to or larger than 9.6 and post-treatment score below 11.3), reliable change (RC; a decrease in scores equal to or larger than 9.6), no RC (a decrease or increase in scores <9.6) and deterioration (an increase in scores equal to or larger than 9.6). In the total sample of the RCT (*n* = 94), 31.9% (*n* = 30) of the patients changed clinically significant, 20.2% (*n* = 19) changed reliably, 23.4% (*n* = 22) remained unchanged and 3.2% (*n* = 3) deteriorated in scores on the BDI-II-NL; 21.3% (*n* = 20) had missing outcome data (see Figure 1).

### Participants

For the current study, patients showing no reliable change in pre-to-post outcome scores on the BDI-II-NL (van der Does, 2002) were included. We did not incorporate deteriorated patients based on the assumption that non-improvement and worsening are distinct phenomena with potential different clinical implications (cf. supra; Mohr, 1995; Lambert, 2011). For the same reason, we excluded patients who ended treatment prematurely (i.e., drop-out from treatment), which was defined as the patient-initiated premature termination of therapy within four sessions of treatment (in line with other commonly used definitions of drop-out; Wierzbicki and Pekarik, 1993; cf. Barrett et al., 2008) This resulted in the selection of 19 participants. The flowchart in Figure 1 gives an overview of the selection process for this study. The sample consisted of 12 women and 7 men ranging in age from 21 to 59 (*M* = 34; *SD* = 10.7). All patients were born in Belgium except for 1 patient who was born in the Netherlands; 1 patient had a parent of foreign origin. Table 1 gives an overview of the demographic information per patient. During the study, 8 patients received CBT; 11 patients received PDT. Patients' average treatment duration was 17 sessions (range 6–20 sessions). All patients were diagnosed with major depression prior to treatment (comorbid Axis I diagnoses as assessed using the SCID for DSM-IV-TR are presented in Table 1).

**Table 1:** Demographical information of patients in the sample.

**Pt**	**M/F**	**Age range**	**Marital status**	**Education level**	**Employment status**	**Therapy (*n* sessions)**	**Prior care**	**Comorbid diagnoses**
A	F	35–40	Divorced	Higher	Employed	PDT (20)	Therapy	Panic disorder
B	F	25–30	Single	Higher	Internship	PDT (20)	No	GAD
C	F	35–40	Cohabiting	Higher	Employed	PDT (20)	Both	OCD; GAD
D	M	35–40	Single	Secondary	Interrupted	PDT (20)	No	None
E	F	55–60	Married	Secondary	Employed	PDT (20)	No	OCD; Pain D.; ED.
F	M	50–55	Married	Higher	Unemployed	PDT (20)	Therapy	None
G	F	50–55	Divorced	Higher	Housewife	PDT (7)	Meds	Somatisation D., BDD
H	F	30–35	Single	Secondary	Unemployed	PDT (20)	Both	Agoraphobia; BDD
I	M	20–25	Single	Secondary	Student	PDT (20)	No	Panic disorder; GAD
J	F	25–30	Single	Secondary	Interrupted	PDT (20)	Both	Social Phobia; GAD; ED
K	M	25–30	Single	Higher	Unemployed	CBT (20)	Therapy	None
L	M	30–35	Single	Secondary	Unemployed	CBT (12)	Both	Specific phobia; OCD
M	F	20–25	Cohabiting	Secondary	Employed	CBT (6)	Both	None
N	F	25–30	Single	Higher	Employed	CBT (20)	Therapy	PTSD
O	F	20–25	Cohabiting	Higher	Student	CBT (17)	Both	GAD
P	F	50–55	Divorced	Secondary	Employed	CBT (20)	Both	None
Q	M	35–40	Cohabiting	Higher	Employed	CBT (8)	No	None
R	M	40–45	Single	Higher	Employed	CBT (20)	Both	Panic Disorder
S	F	25–30	Cohabiting	Higher	Employed	CBT (20)	Therapy	Panic D., Agoraphobia; Social phobia; OCD; GAD; PTSD; Hypochondrias

*Information as indicated prior to therapy. To safeguard participants' anonymity, no exact ages are mentioned in the table. M/F: male/female. “Cohabiting”: living together with romantic partner. “Higher education”: college or university degree. “Interrupted employment” (i.e., temporarily): e.g., due to sick leave. “Prior care”: previous psychotherapy or medication (i.e., antidepressants or other psychopharmaceutic treatment). “Both”: medication and psychotherapy. GAD, generalised anxiety disorder; OCD, obsessive compulsive disorder; ED, eating disorder; BDD, body dysmorphic disorder; PTSD, post-traumatic stress disorder*.

### Grounded Theory Analysis

Grounded Theory (Glaser and Strauss, 1967) can be described as an explorative and interpretative qualitative research method, aimed at the construction of new theories or rationales grounded in data (in our case patient interviews) (Fassinger, 2005; Charmaz, 2014). Using this method, a tentative conceptual model of non-improvement that comprises patients' experienced changes and explanatory factors was created Characteristic of grounded theory, several stages of analysis were completed in a cyclic manner before arriving at the final conceptual model (Mortelmans, 2013). This form of inquiry enabled the exploration of the phenomenon of non-improvement in the participants' terminology and to identify themes in the data in a bottom-up manner. As the interviews were conducted in the context of a larger study, the interview questions were not altered throughout the data gathering process as is often the case in grounded theory analysis.

Prior to the actual coding of the interview transcripts, the first author wrote a vignette about every participant that included demographic information, treatment duration, pre-post outcome scores and a summary of the most important themes addressed in the interview. The vignettes were used to get an initial idea of the individual cases in the sample prior to the analysis. During later stages, the first author repeatedly reread the vignettes to validate the constructed model and conclusions with the individual cases. The interview transcripts were subsequently analysed by the first author in dialogue with the third author; the second author functioned as an auditor throughout the process (Hill et al., 1997).

#### Open Coding

Open coding is defined as “the analytic process through which concepts are identified and their properties and dimensions are discovered” (Strauss and Corbin, 1990, p. 101). In this phase, the interviews were first read and reread to identify relevant parts of the interviews relating to the research questions (cf. selecting meaning units; Giorgi and Giorgi, 2003). Labels were attached to certain parts of the text, differentiating experienced changes/remaining issues from therapy factors and other mentioned influences. Non-relevant parts, i.e., not dealing with the topic of well-being, experienced changes or therapy, for instance, were omitted. In order to prevent relevant information from being omitted during the coding process, this work was first conducted on printed versions of the interview transcripts and repeated in the Nvivo software package. This phase resulted in a first list of codes that were formulated with the intent to remain close to the narrative of patients. A first rough classification was made between the various codes (i.e., experienced changes, remaining issues, therapy effects, social context). They were discussed between the first and third author and altered until consensus was reached.

#### Axial Coding

Axial coding can be summarised as “the process of relating categories to their subcategories termed ‘axial' because coding occurs around the axis of a category, linking categories at the level of properties and dimensions” (Strauss and Corbin, 1990, p. 123). In this phase, the various codes were further divided into subcategories in order to refine the first initial classification of codes. In dialogue between the first and third author, the resulting codes were thematically connected and where needed rephrased. At the end of this phase, the first author looked for visual images and metaphors that could help to grasp the central categories and mechanisms emerging from the narratives of the patients (e.g., “stuck in a maze”; “impasse”). These were further developed and refined in the next phase.

#### Selective Coding

Selective coding comprises “the process of integration and refining the theory” (Strauss and Corbin, 1990, p. 143). In this phase the theory was cultivated by creating a core category and building other categories around it. In discussion with the third author, this theory was refined. The second author audited the selection of the core and subcategories by asking critical questions regarding the rationale behind the extracted central mechanism. At the end of this phase a set of subcategories was created based on the entire nonimproved sample. Subsequently, we looked into the frequencies of the different categories represented in the CBT and PDT group in order to unravel therapy-related differences. These were included in a detailed table. After finalising the theory, adequate and valuable phrases were chosen to describe the categories and informative quotes were selected to illustrate the various categories and their interrelations and influences. Patients were given a letter of the alphabet to anonymise text fragments (i.e., from A to S).

#### Credibility

Credibility checks were held at several stages of the analysis. At the end of every interview, patients were asked whether they wanted to add further information that had not been addressed in the interview. During the analysis, we tried to remain transparent about the entire process (Stiles, 1993) and we acknowledge the influence of the perspective and background of the researchers. The researchers' personal interest in patients' idiosyncratic perspective for instance instructed the focus of the study and analysis. Potential consequences of implicit guiding assumptions were controlled as much as possible by making this idiosyncratic focus central to our study (Creswell and Miller, 2000). We furthermore departed from the assumption that “non-improvement” can also include changes, therefore this was explicitly integrated in our research questions. We worked in a systematic manner to form conclusions and interpretations (Stiles, 1993) and attempted to stay open for any information coming from the narratives throughout the entire process. The analysis aimed at outlining macro-processes in psychotherapy, i.e., examining a wide angle rather than micro-processes (e.g., specific therapeutic effects) and investigated the subjective experience of several different participants (i.e., between-case variation) (Denzin and Lincoln, 2005). In line with our research aim to investigate therapy and outcome in a broader context, the analysis and interpretation of patients' narratives were conducted using a contextual perspective that departs from the assumption that the broader social context influences how patients give meaning to their experiences (Boyatzis, 1998). Triangulation among researchers, several interviews, and quantitative and qualitative indications of outcome were applied to gain different perspectives on the issue. The ultimate themes were formed by asking critical questions regarding codes and categories (Mortelmans, 2011).

## Results

In this section, we will present the quantitative pre-post outcome data and qualitative analysis of patients' experiences respectively. Interpretations of the quantitative and qualitative findings will be described separately; broader integrative conclusions and implications will be presented in the discussion.

### Descriptive Pre-post Outcome Scores on the Beck Depression Inventory

Table 2 summarises the average score on the BDI-II-NL (Beck et al., 1996; van der Does, 2002) before and after treatment, the standard deviations (SD) and range in scores (i.e., minimum and maximum score) for the entire nonimproved sample, the PDT and CBT group.

**Table 2:** Pre-post outcome scores on the BDI-II-NL.

**Total score (BDI-II-NL)**	**All** ***n*** **=** **19**		**PDT** ***n*** **=** **11**		**CBT** ***n*** **=** **8**	
	**M**	**SD (range)**	**M**	**SD (range)**	**M**	**SD (range)**
Start therapy	30	5.3 (22–42)	31	10.2 (24–36)	29	6.2 (22–42)
End therapy	30	7.8 (18–46)	29	7.4 (20–44)	30	8.3 (18–46)

*Meaning of scores on the BDI-II-NL (van der Does, 2002): 0–13: minimal depression, 14–19: mild depression, 20–28: moderate depression, 29–63 severe depression. Cut-off clinical range: 11.3*.

Both at the start and end of therapy, the non-improved patient group is characterised by a wide range in scores. At the start of treatment, patients scores varied between moderate depression (*n* = 10) and severe depression (*n* = 9) (cf. van der Does, 2002). At treatment termination, 1 patient scored mildly depressed, 9 scored moderately depressed and 9 others scored severely depressed. The average score both before (30) and after (30) therapy indicate severe depression for the total sample, although at the borderline of moderate depression. All patients remained in the clinical range and did not change reliably in scores compared to the start of treatment.

### Conceptual Model of Non-improvement From Depressed Patients' Perspective

**Figure 2:**
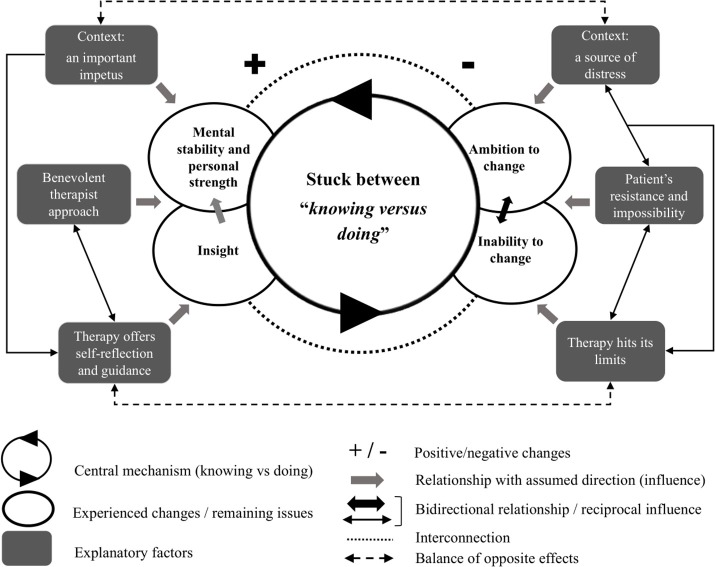
Conceptual model of non-improvement from depressed patients' perspective.

The grounded theory analysis of nonimproved depressed patients' narratives resulted in the core category *Stuck between “knowing vs. doing*.” Around this core category, a model was constructed consisting of 10 subcategories that help to explain this core concept. The subcategories are divided into the changes and remaining issues patients mentioned and the positive and negative influences patients ascribed these changes/remaining issues to. These influences are referred to as “explanatory factors” and specified as “facilitating factors” and “impeding factors.” Figure 2 depicts the conceptual model: The left part of the model comprises positive changes and facilitating factors, the right part of the model shows remaining issues and impeding factors. Table 3 summarises all core and subcategories in more detail for the entire nonimproved sample, the PDT, and CBT group. The frequencies of patients contributing to each category were added.

**Table 3:** Taxonomy of non-improvement based on depressed patients' perspective.

**Nonimproved outcome**	**All**	**PDT**	**CBT**
**Core category** *Stuck between “knowing vs. doing”*	*n = 19*	*n* = 11	*n* = 8
**Positive changes and facilitating factors**	***n***	***n***	***n***
Positive changes	*16*	*9*	*7*
Mental stability and personal strength	14	9	5
Insight	15	9	6
Facilitating factors	*18*	*10*	*8*
Therapy offers self-reflection and guidance	*14*	*10*	*4*
Talking and letting it all out	6	5	1
Reflection leading to insight	11	7	4
Provided insight and practical help	12	7	5
Benevolent therapist approach	*17*	*9*	*8*
The right questions	9	8	7
Good relationship	16	9	7
The context as important impetus	*11*	*6*	*5*
**Remaining issues and impeding factors**			
Remaining issues	*18*	*11*	*7*
Ambition to change	13	9	4
Inability to change	13	8	5
Impeding factors	*19*	*11*	*8*
Therapy hits its limits	*19*	*11*	*8*
Something missing	12	8	4
Mismatch and doubt	17	10	7
The patient's:	*18*	*11*	*7*
Resistance	16	9	7
Impossibility	12	9	6
The context as source of distress	*16*	*9*	*7*

*All patients mentioned multiple experienced changes, remaining issues, and explanatory factors. The indicated frequencies near the categories show this multidimensionality and subcategories thus do not add up. The maximum frequency per category is 19 for the total sample, 11 in PDT and 8 in CBT. Italics indicate the number of participants contributing to overarching themes*.

#### Core Category: Stuck Between “Knowing vs. Doing”

The central mechanism for understanding non-improvement from the patients' perspective is “knowing versus doing”: A feeling of having acquired certain changes yet being unable to go a step further, or to know what the problem is but feeling an incapacity to do deal with it. In general, patients wanted to move forward but felt unable to. Some patients stated this literally: “Rationally I know what my problems are or what I should do, but there is just nothing changing.” At the same time, the core category captures the effects of therapy that on the one hand facilitated patients' self-understanding and mental stability, but on the other have not been able to overcome certain barriers. A plus (for positive changes and positive influences) and minus (for remaining issues and negative influences) seem to cancel out each other, resulting in a stalemate.

“I have learned a lot, I have gained many insights. (…) I am not as despondent anymore, but if I say that ‘not that much has changed' I mean, I still have difficult periods and a few fundamental problems, which I do understand better now, are not really solved yet, or maybe they are not easy to solve. So, I know much more, I have improved on the level of knowing, but not so much on the more practical level.” (Patient C., CBT)

#### Positive Changes

The overarching experience of being stuck does not imply that patients have not experienced changes at all. Two themes resulted from the analysis showing that throughout the process of therapy, patients have grown *mental stability and strength* (*n* = 14) and have gained more *insight* (*n* = 15). Moreover, these changes seem interconnected, as increased understanding was said to have influenced patients' personal strength. In the model in Figure 2, this is indicated by a dotted line (showing interconnection) and an arrow from “Insight” to “Mental stability and personal strength.”

“In general, I feel much stronger, mentally. I have gained many insights in therapy. (…) It gave me peace of mind and recognition that okay, my thoughts, experience and things I long for are not that strange.” (Patient J., PDT)

##### Mental stability and personal strength

Increased mental stability (*n* = 11) consisted of two subthemes: A more positive state of mind (*n* = 9) and the ability to accept and let things go *(n* = 6). Patients had learned to deal with certain situations, they felt less emotionally overwhelmed and believed they could handle challenges better. Patients' personal strength (*n* = 11) consisted of an increased self-confidence (*n* = 11) and being more vigorous (*n* = 9). Patients felt more active, dared to socially interact and felt less anxious and insecure.

Interviewer: “So you say you are mentally not dispirited anymore. Can you explain the difference with before?”

Patient: “I think that before I was really struggling with myself, my self-image and now I feel more balanced. I can take a distance. The fights [with partner] are still intense, but I relate it to the relationship now, I've stopped blaming myself.” (Patient Q., CBT).

##### Insight

Increased insight was described on three levels. Patients were able to see things from a different perspective (*n* = 9), understood themselves better (*n* = 8) and had gained insight into the reasons why they experienced difficulties (*n* = 8).

“Therapy made me reflect upon things and gave me some different ideas about situations that were clear to me but might not have been that clear after all or that needed to be looked at from a different perspective.” (Patient A., PDT).

“I have learned more about myself. I already knew I was stubborn, but we talked about it a lot [in therapy], about how I set my standards very high and I don't accept help from anyone and that, by this, I make life very difficult for myself.” (Patient H., PDT).

#### Facilitating Factors

The facilitating factors contributing to these positive changes according to patients include the role of therapy, the therapist and the patients' social and professional context. As indicated in Figure 2 by unidirectional arrows, these features were described as contributing to patients' mental stability and strength as well as insight.

##### Therapy offers self-reflection and guidance

Patients in CBT and PDT presented slightly different experiences of therapy. These differences seem in line with the specific nature of both types of therapy, given the explorative and expressive style of PDT and the directive approach in CBT. Especially in the PDT group, weekly therapy sessions were considered important for being able to talk, express feelings and thoughts (*n* = 6) and therapy was seen a as a weekly moment of self-reflection (*n* = 11). This seemed to have stimulated both patients' insight (cf. self-reflection) and mental stability and strength (e.g., letting things out; relief).

“By saying things out loud in therapy, you start reflecting on them and it becomes a reality that does not only exist in your head. We had one session where the therapist asked some questions about my relationship and because I really did not want to answer them, I started wondering, how I really feel in this relationship, I realised that wasn't very good.” (Patient B., PDT).

In the CBT group, similar aspects were mentioned (*n* = 4), but therapy was also valued for actively providing patients with insight and practical help (*n* = 5), making the facilitating role of therapy more guiding than reflective.

“She [the therapist] helped me to take certain steps. We tried to come up with means to…, like a priority list for my day, because it is difficult for me to…, although I know I have to do lots of things, I'm not organised.” (Patient R., CBT)

##### Benevolent therapist approach

Patients in both therapy groups ascribed important changes more to the therapist's way of being than to the therapy form as such. Therefore, we explicitly describe therapy and therapist separately, even though the factors are clearly intertwined. This reciprocity is indicated in the conceptual model (Figure 2) by means of a bidirectional arrow. Firstly, a *good therapeutic relationship* was described by the majority of patients (*n* = 16) as making it easier for them to talk and open up. More specifically, patients valued that the therapist did not judge but rather encouraged and acknowledged them (*n* = 10). Secondly, the therapist had the skill to ask *the right questions* (*n* = 15) that stimulated reflection and provided patients with a different perspective. The previously discussed themes “insight” and “mental stability” were described as influenced by this approach of the therapist. Although the therapist was mentioned as important in both CBT and PDT, the nature of the experienced role of the therapist's different: In PDT, the therapist was described as stimulating a mental process that was ongoing for the patient, while the CBT therapist was characterised as being an active participant or initiator in the therapy sessions.

“His questions, they often appeared so innocent, but when you think about it afterwards you see things from a different perspective. Very subtle because he does not tend to give his own opinion.” (Patient C., PDT).

“After a few sessions, my therapist came up with a scheme that summarised my life until now and where my anxiety comes from. I remember I had to cry for the first time. I noticed how good it felt to finally be understood. I still believe he can help me in the process of accepting it [certain life events].” (Patient S., CBT).

##### The context as an important impetus

Besides therapy and the therapist, patients mentioned the influence of their social context as a third facilitating factor. Significant others appeared as an important motivation to do something about problems, to find a job or to keep on going (*n* = 11). For some patients, it had been important to be at home for a while (with sick leave) and to have the time for themselves in their own space (*n* = 10). For others (*n* = 5), work (i.e., the professional context) was an important support mechanism, as it gave them a reason to get up in the morning and structure to their day. The motivating context was therefore considered a facilitating factor for patients' mental stability and personal strength and seemingly a potential stimulus for engaging or continuing treatment.

“My son is one of the reasons for starting and continuing the course [i.e., education]. I want to be able to show him something, instead of being an unhappy person. I want to give him something, something positive. That's actually the only valuable thing in my life that's left. I used to be so materialistic, now the only thing that matters is him.” (Patient L., CBT).

#### Remaining Issues

Despite the positive changes in mental stability, personal strength and gained insight, certain issues remained. Feeling stuck was characterised by the wish or ambition to change yet feeling unable to do so. A reciprocal arrow between the categories “Ambition to change” and “Inability to change” represents this equilibrium.

##### Ambition to change

The majority of the patients experienced an ongoing struggle and carried hopes for further change (*n* = 13). These aspects implied things they believed they still lacked or they should work on in the near future, such as tackling self-criticism and self-discipline.

“I wish I could have a more positive stance in life, to be able to counter my negative thoughts. I want to have the discipline to get things done, but the hours just slip away, I see the days pass by without getting anything done. That's wasted time to me. I want to get a grip of it.” (Patient I., PDT)

##### Inability to change

In spite of and seemingly in conflict with the ambition to change is an overall feeling of inability (*n* = 13). Patients felt as if they were running behind on things, they lacked initiative and could not force themselves to move forward.

“I'm just not on top of things. I constantly feel rushed, but I lose so much time. Searching for things, not able to finish things because you are too distracted. I just can't seem to get out of it. Things pile up, it seems that for every problem I solve I get two in return” (Patient P., CBT)

Some patients described an inescapable cycle they seemed to repeat over and over again (*n* = 3). This inability was also reflected in what seemed like an internal conflict (*n* = 7), for instance, being stuck in the struggle between wanting to spend more time with the kids but at the same time aspiring for a professional career. One patient was highly preoccupied with a long-lost dream, which made it impossible to pursue a new goal in life.

#### Impeding Factors

Similar to the factors that help explain positive changes, patients brought up potential reasons for why certain issues remained unchanged. These factors include the limits of therapy, the role of the patient and a negative influence coming from patients' social context.

##### Therapy hits its limits

Notwithstanding its positive effects, therapy was described as hitting a limit by all patients. More specifically, advise or learned techniques were considered valuable, yet only up to a certain point. Patients stated that they were unable to use the techniques when feeling really bad, or they did not find the time to do so. For others, therapy had progressed too slowly, had not been valuable every session, or it had worked for some aspects but not for others. Therapy hit a limit in two ways: *Something was missing* in therapy (*n* = 12) and/or the therapy *mismatched* the patient's needs or expectations at some point (*n* = 17). Noteworthy is the observed difference between the PDT and CBT group. A few patients in PDT were displeased that they had not gained the right tools, were not given any directions (*n* = 4) or stated therapy had a varying impact (sometimes it helped, sometimes it did not) (*n* = 3).

“I expected her [the therapist] to give me good advice on how to deal with my problems. How I can worry less, how I can improve my breathing, just some tips. I must have imagined therapy the wrong way, she did not give me any tips. I'm kind of disappointed. (…) I still don't understand the purpose of talking about all these things, I often felt worse after the session.” (Patient G., PDT).

Some patients in the CBT group, on the other hand, stated therapy was too superficial and they needed a more intense form of treatment (*n* = 5).

“The first three to four sessions, you tell your whole life story and all that is said about it is just okay, ‘you suffer most from the discussions [at home/with your partner] so let's see how we can handle them.' While I thought okay, I just told you my entire life story, about who I am and how I became who I am, that could have been included in therapy, but I actually felt that it wasn't at all, we just looked at one segment.” (Patient Q., CBT).

Moreover, therapy as hitting its limits seems to have contributed to a rather ambivalent attitude toward possible continuation of therapy (*n* = 11). One third of the patients had no further need or motivation to continue psychotherapy (*n* = 8). Some of them believed they had dealt with everything or had gotten everything out of therapy that they could, others had lost hope that therapy could help them or were disappointed about the results. Half of the patients indicated they would continue the same therapy, because they felt committed to the process (*n* = 6) or because they had further specific issues they wished to address (*n* = 4). Others, however, were interested in pursuing a different kind of therapy (*n* = 7).

##### The patient's resistance and impossibility

Patients also reflected on their own role and position in therapy. Most patients described feeling a certain resistance toward therapy (*n* = 16). For instance, they did not take therapy very seriously, had difficulties with opening up or were reluctant to do certain exercises. Several patients saw therapy as a task or an investment that asked too much from them (e.g., energy, time, money) (*n* = 6). This rather ambivalent position in therapy was, for instance, described by patient R:

“I was afraid to fail in therapy. (…) I typically start things but can't manage to continue them. Maybe because (…) when it hasn't gone well one day, I can't let that go. It is all or nothing often, so I was afraid I would not do very well [in therapy] and also, sometimes I put effort into it, but often I was busy doing other things I thought I should be doing, like work.” (Patient R., CBT).

Secondly, many patients were convinced about the fundamental nature of their problems and the impossibility to change (*n* = 15), sometimes referring to their own personality. Moreover, some patients indicated that therapy of 20 sessions is in general too little to solve more fundamental problems. The patient's resistance and idea of impossibility seem to correspond to the perspective on therapy as hitting a limit. In Figure 2 this was indicated by means of a reciprocal arrow, assuming a certain reciprocity between both explanatory factors.

“I think I'm quite a different case, I have quite a big tendency toward depression, if I compare myself to other people in my environment who have depression, they get over it after a year, but I think for me this is a bit more difficult, because of my childhood…I have been conditioned to think in a certain way, I think that matters a lot [for the duration and effect of therapy].” (Patient O., CBT).

Despite a mismatch, some patients described they were able to get passed initial resistance and adjusted to therapy, while for others, the limits of therapy, their own resistance or feelings of impossibility lasted throughout the therapy process.

“I might have expected therapy to be a bit more practical, but I noticed quite fast that this wasn't the goal of the sessions and I accepted that, I didn't see the talking as a waste of time” (Patient I., PDT).

“So, I think ‘okay, therapy has ended now and *once again* I'm nowhere, it did not help, and it only cost me money, a lot of time and energy, and why? For nothing.' (…) Of course, I know I did make progress, but I it's hard for me to see it that way. I just think, ‘it's the umpteenth thing I've tried and what is the use?”' (Patient P., CBT).

##### The context as a source of distress

Several contextual factors were mentioned as having a negative influence on patients' wellbeing during and after therapy. Firstly, patients' personal context was mentioned as being highly stressful (*n* = 16). Several patients, for instance, encountered a conflict with family members (*n* = 6). These difficulties in relationships were often considered to facilitate or perpetuate certain problems, and consequently they were believed to have influenced the therapy process and patients' progression. Patient F., for instance, described a critical moment in course of therapy:

“There was a crisis [during treatment]. It was when I just started working there [family business], it became too much with how my brother-in-law always got angry with me. I could not handle it. He yelled at me that I was making him bankrupted, I cost a fortune, I don't work well, I'm too slow. At a certain point I switched off my phone and just ran away, I wanted to disappear, commit suicide.” (Patient F., PDT).

Secondly, the professional context appeared as a source of stress or dissatisfaction (*n* = 9). This subtheme contains patients who experienced high amounts of stress due to school-related deadlines, had difficulties adjusting to a corporate culture (e.g., not able to handle the given freedom) or did not experience any fulfillment at work. Finally, many patients mentioned external factors causing distress, like certain events or circumstances (*n* = 12), for instance, dealing with unexplainable physical complaints and an ongoing lawsuit. The reciprocal arrow in the conceptual model between the patient's resistance and impossibility and stressful context indicates that both factors are understood as interacting and influencing the therapy and recovery process.

## Discussion

This study investigated the phenomenon of non-improvement in psychotherapy from the perspective of depressed patients in relation to their pre-post outcome scores showing no reliable change. By doing so, we answered the pressing need for further investigation of the phenomenon of negative outcome and the exploration of the relationship between quantitative and qualitative approaches to outcome and treatment evaluation in the field of psychotherapy (McLeod, 2013).

First and foremost, the findings of this study showed that non-improvement as indicated by symptom-based outcome scores did not mean that patients did not experience any changes. Where a lack of change in outcome scores means a status quo on the level of symptom-severity, the interviews of the patients revealed a more nuanced and complex picture. Central to patients' experience of non-improvement is the mechanism of knowing vs. doing. While patients had the ambition to change, they felt unable to overcome certain problems, resulting in a stalemate of knowing what to change but not being able to. Positive changes were offset by substantial remaining issues: Increased mental stability, personal strength and insights were gained, yet these did not result in changes on other levels of patients' lives. From the patients' perspective, “no change” in symptom-based outcome scores seemed to be “not enough” change or a “partial change.” The therapy, the therapist, the patient and context facilitated positive changes but at the same time were unable to alter important issues or even impeded patients' progression (resulting in remaining issues). None of these factors can be considered the main or only explanatory reason but must be understood as interacting (cf. Mash and Hunsley, 1993; Werbart et al., 2015). In sum, an equilibrium between a positive and negative pole seems to characterise the depressed patients' experience of non-improvement.

A similar positive-negative balance has been observed by Werbart and colleagues in a study on non-improved patients' experience of psychotherapy (2015). Nonimproved patients perceived their therapy as “spinning one's wheels”: Therapy was valued for some aspects but disappointing on others and even though some changes occurred, core difficulties remained. The current study investigated nonimproved patients' experiences of outcome and therapy in a broader context of various potential explanatory factors (i.e., not limited to the effects of therapy). In that sense, the findings of this study and the study of Werbart et al. (2015) can be seen as complementing each other, also because different populations of patients were investigated (i.e., adults and young adults). Notably, the experience of both outcome and therapy are strongly congruent in reflecting a balance between a plus (i.e., positive changes and facilitating factors) and minus (i.e., negative changes/remaining issues and impeding factors).

The positive pole of the resulting conceptual model in this study, including increased mental stability, personal strength and insight, corresponds to findings of other qualitative outcome studies. Mental stability and personal strength relate to what has been described as feelings of empowerment and improved emotional functioning (McElvaney and Timulak, 2013), and more generally, as changes on the level of the self (Timulak and Creaner, 2010). Strikingly and in contrast to findings from studies on patients' experience of positive outcome, our nonimproved sample did not report changes on an interpersonal level (Nilsson et al., 2007; Binder et al., 2009; Timulak and Creaner, 2010); reported positive changes were overall more self-focused. Indeed, we could wonder whether improvement on a symptomatic level enables or coincides with changes on a more interpersonal level. Regarding patients' gained insight, our findings seem partially in contrast to the commonly derived conclusion that insight is an important acquisition for obtaining positive outcome (see the recently published meta-analysis of Jennissen et al., 2018). In this study, increased insight facilitated patients' mental stability and personal strength, but it did not alleviate patients' self-reported symptoms or alter core difficulties. Similarly, Lilliengren and Werbart (2005) found that self-knowledge does not always coincide with changes in underlying problems. Qualitative studies suggest an important role for agency regarding this link between insight and outcome: Rather than gaining insight as such, it is important to gain the capacity to apply or act upon gained insight in daily life (McLeod, 2013)^3^. Stage models of therapy (see for instance Hill, 2004), state that insight is only valuable to the extent that it leads to action. The absence of this active component could explain the lack of improvement on other levels in our research sample. As already stated by Freud, in order to gain substantial change, a step beyond intellectual insight toward experience might be required (see Bohart, 1993; Castonguay and Hill, 2007). More research on the mechanism of how insight promotes change is, however, warranted.

The helping role of treatment differed depending on the type of therapy. In accordance with the finding of Nilsson et al. (2007), patients valued CBT and PDT for different reasons. In our study, therapy provided a moment of self-reflection for patients in the PDT group, while practical help and guidance was valued in the CBT group. Interestingly, while patients in both CBT and PDT mentioned the central role of the therapist, its specific effectuation differed seemingly. In line with the differentiation between the approach of the respective therapies, the PDT therapist was attributed a rather subtle though powerful technique stimulating reflection in patients. The CBT therapist, on the other hand, was considered an active participant in treatment who offered patients insight via tools such as schematic overviews. A good therapeutic relationship was one of the most important common factors in psychotherapy (Lambert and Barley, 2001) mentioned by the majority of the patients in our study, similar to other qualitative findings (Levitt et al., 2016).

Nonetheless, all patients stated therapy hit a certain limit. Again, in line with the observation of Nilsson et al. (2007), both types of therapy were criticised on a different basis: In our sample, dissatisfied patients criticised CBT for being too superficial while PDT was criticised for not offering the right tools or direction. The latter corresponds to findings from the study of Lilliengren and Werbart (2005), in which patients experienced similar disappointments in psychoanalytic therapy (e.g., wanting a more active therapist, guidance, feedback, and advice). A possible mismatch between certain patients' needs or expectations and the type of therapy supports the increasing emphasis in research to explore which type of therapy works best for whom and focus on the tailoring of treatment to patients' transdiagnostic characteristics (Norcross and Wampold, 2011).

Beyond therapy hitting limits, patients in this study mentioned explicitly that they themselves encountered a certain resistance or hit their own limits and limitations. The patient's in-therapy behavior, such as client involvement and motivation, is the single most important predictor of outcome. Patient motivation has moreover been linked to expectations and hopefulness: Patients who do not believe they can change, and who feel hopeless, may have less motivation to participate in therapy (Bohart and Wade, 2013). Accordingly, the participation in the therapy process seemed rather ambivalent in our sample. Notably, individual differences were observed: Some patients were able to get passed initial doubt about the therapy approach and their own ideas of impossibilities, while others did not. Although not mentioned by patients themselves, it is important to consider that many patients in the sample presented with one or more comorbid disorders, in most cases some kind of anxiety disorder at the start of therapy. Previous research has shown comorbidity in general predicts worse outcome (see Lambert, 2013).

Finally, our findings revealed the therapy process was intertwined with influences from outside the therapy room. Patients' personal context was both considered an important motivation as well as a large source of distress. Again, opposite effects facilitated and impeded changes. It has been outlined that the context plays a central role in sustaining involvement in psychotherapy or undermining this effort (Lambert, 1992; Drisko, 2004). The impact of patients' professional context on their well-being mentioned in this study is in line with robust findings on the impact of job satisfaction on mental health (Faragher et al., 2005). Whilst most qualitative studies tend to focus specifically on patients' experience of psychotherapy, our study provides a valuable additional element of contextualisation.

The resulting negative pole of the conceptual model of non-improvement, including the ambition yet inability to change, shows resemblance to what is considered a central characteristic of experiencing depression: Running behind on things, lacking initiative and motivation (DSM-5; APA, 2013). Feelings of hopelessness and helplessness were unresolved, in line with the remaining average score of severe depression in the sample. However, the question should be posed whether this feeling remained unaltered or rather emerged throughout the process of therapy, for instance, as a response to a lack of improvement. A pre-post research design, even when including retrospective inquiry of patients' experiences prior to treatment, falls short in answering this question. Longitudinal research that includes patients' experiences at the start of therapy as well as monitors changes throughout the process of therapy is needed (cf. De Smet and Meganck, 2018).

The contextual model of psychotherapy as described by Wampold and Imel (2015), offers a valuable framework for interpreting our research findings. In this model, therapy is perceived as a “socially imbedded healing practice” (p. 258) in which the relationship between the therapist and patient is central. According to this model, three pathways lead to change in patients' wellbeing: The first pathway establishes the personal relationship (“real relationship”) between patient and therapist, characterised by genuine interest and empathy, the second pathways creates expectations in patients of being able to overcome their difficulties, and the final pathway includes therapy specific features or tasks. Although all three pathways lead to a certain degree of change, central for the therapy to work is that it can engage patients to follow the treatment rationale and overcome personal beliefs and explanations for distress.

Regarding the first pathway, the contextual model assumes that establishing a real relationship with the therapist leads to general well-being rather than symptom reduction (Wampold and Imel, 2015). Correspondingly, we observed an increased mental stability and personal strength while patients remained unchanged on a symptomatic level. Patients' pessimistic expectations regarding improvement moreover remained unaltered and for many it was difficult to adapt to or engage in specific therapy features; the second and third pathway thus seem not (entirely) fulfilled. The dyadic concept of the patient-therapist working alliance (Bordin, 1979) further demonstrates how “the collaborative purposive work” (Hatcher and Barends, 2006, p. 293) was obstructed by this discordance between patient and therapist or therapy; both by therapy not being able to meet patients' need, as well as by patients' resistance toward the requirements of therapy. This links up to the differentiation between two types of bonds: The work-supporting bond and personal relationship (cf. real relationship). The latter involves affective attachment, liking, trust and respect. The other type of bond is considered necessary for “the difficult work” in therapy, for instance dealing with affective or painful material or executing assignments like exposure and homework (Bordin, 1979). This distinction helps to understand how, in our study, patients experienced a good therapeutic (or real) relationship but failed to engage in the work-supporting bond. Being at ease in therapy and feeling accepted and understood by the therapist thus seem important, for instance leading to increased well-being (Wampold and Imel, 2015), mental stability and personal strength, though not enough to facilitate further life-changes. Correspondingly, gaining insight or self-understanding as such might not be enough when “the hard work” of dealing with affective material has not been worked through. While the contextual model ascribes most of the responsibility to the therapist, the current study and findings give more weight to the role of the patient and his personal context (Wampold and Imel, 2015).

These findings yield a number of clinically relevant implications. Patients who find themselves stuck between knowing versus doing, may hit a certain limit due to a mismatch with the therapy offer, experiencing personal resistance or encountering difficulties outside of the therapy room. As these implications may be brought about by (idiosyncratic) underlying reasons, it may be worthwhile to take this as a particular clinical focus. In light of the increasing use of routine outcome measures in clinical care (see Boswell et al., 2015), a lack of changes in symptom severity could indicate any of these reasons and most likely a combination, yet monitoring instruments clearly require further exploration in dialogue with the patient. However, signs of non-improvement may not always be visible for the therapist. Studies have shown that therapists tend to underestimate negative outcome, as patients tend to keep dissatisfaction about therapy to themselves—possibly because they do not want to offend the therapist (McLeod, 2013; Werbart et al., 2015). Therefore, it may be implicated to work on meta-communication in therapy, to avoid or restore possible ruptures in the therapeutic work and relationship (von Below and Werbart, 2012). On the other hand, a well-established therapeutic relationship could change dissatisfaction about therapy into a negotiation, that is, an active focus point in therapy (cf. Wampold and Imel, 2015). In some cases, referring patients to a different approach that is more in line with patients' own rationale may be warranted (Wampold, 2007), as what may work for 1 patient, might not work for the other (Norcross and Wampold, 2011). Also, the optimal duration of treatment may differ among patients. In the current study, the number of sessions was fixed at twenty, which may have been too little to facilitate changes for some patients (e.g., the average “good enough level” has been estimated at 26 sessions; Barkham et al., 2006). Moreover, patients showing high levels of resistance in therapy may benefit from a less directive approach (see Beutler et al., 2002, for an overview of the literature on resistance) in which therapy is adjusted to patients' own pace. This is supported by authors who warn that uniform time limits for treatment may not adequately serve individual patients' needs (Baldwin et al., 2009).

This study addresses the critical concern about misrepresentation of patients' outcome by means of standard outcome evaluation and statistical classification (Kazdin, 2008; Hill et al., 2013). First of all, no reliable change in outcome scores seemingly masked the significant changes experienced by patients and does not allow to represent the particular balance between remaining issues and positive changes. Furthermore, considering patients to be a uniform group based on a similar pattern of outcome scores might overlook important individual differences. None of the patients in our sample stated they were cured, although they did vary in the extent to which they experienced improvement and whether they wanted to continue treatment. In our study, the pre-post changes in outcome scores seem to give a rough preliminary indication of patients functioning, while the patients' narratives show non-improvement is more complex and diverse than can be grasped by a lack of symptom reduction (in line with Zimmerman et al., 2006, 2012). This observation is not surprising in light of the complexity and heterogeneity of depression experiences (Ratcliffe, 2014). It is plausible to assume that recovering from depression is at least equally diverse and layered (cf. von Below et al., 2010).

Consequently, the findings of this study shed light on the previously voiced question of how negative outcome and non-improvement should be conceptualised. In general, similar to previous research findings, patients' treatment satisfaction and negative outcome did not show a one-on-one correspondence (Werbart et al., 2015); while all patients stated therapy hit a certain limit, a minority was also clearly dissatisfied. Mash and Hunsley (1993) have argued that “without a guiding theoretical framework for considering failing treatments, the assessment task is daunting, because almost any event in therapy might be construed as a possible indication that treatment is currently failing or is about to fail.” (p. 293). This study shows how this endeavour benefits from a mixed-methods research format that integrates a grounded theory approach. In line with the strengths of grounded theory (Fassinger, 2005; Mortelmans, 2011; Charmaz, 2014), further theory-building research can mean an important contribution here (cf. Stiles, 2015).

### Strengths, Limitations, and Future Directions

The implications of this study address the well-known gap between academic research and clinical practice (Castonguay et al., 2013). RCTs as golden standard research format are limited in providing knowledge that can inform clinical practice (Westen et al., 2004). The value of integrating qualitative research into this type of rigorous research has therefore been emphasised (Midgley et al., 2014). The current study provides an actual example and informs both clinicians and research on the relationship between outcome scores and patients' experiences of non-improvement. It furthermore builds on the literature of helping and hindering therapy features (Paulson et al., 2001; von Below and Werbart, 2012) by placing the experience of therapy in a broader context of potential explanatory factors as mentioned by patients.

The current study is one of few examining the relationship between quantitative and qualitative outcome evaluation of non-improvement (McElvaney and Timulak, 2013; McLeod, 2013). Focusing on this particular subgroup rather than deteriorated or dissatisfied patients allows for the contribution to a lack of specificity in outcome research and the literature on negative outcome (Lambert, 2011). Research suggests non-improvement, deterioration and patient satisfaction do not fully correspond, although they are often used interchangeably (Lampropoulos, 2011). The current study gives an overall conceptual model of non-improvement and potential explanatory factors. Whether this is, however, representative for nonimproved depressed patients cannot be concluded. Further research should focus on investigating differences and similarities between various groups of outcomes (cf. recovery, improvement, no change, and deterioration; Jacobson and Truax, 1991) in order to get a better understanding of the clinical meaningfulness of change from the perspective of patients.

This study contributes to the understanding of non-improvement in psychotherapy and the relationship between quantitative and qualitative outcome evaluation. It cannot, however, answer the question whether outcome scores were representative for every individual patient. The focus of the present study was to provide an overall understanding, (i.e., a conceptual model) of non-improvement relying on a larger group of nonimproved patients. More idiosyncratic information still remains unaddressed and case-study research focusing on individual patients' narratives and outcome scores is warranted (Kazdin, 2011). Similarly, the study cannot offer a fine-grained comparison of the specific effects of CBT and PDT, which could be further addressed in research on specific factors. The mixed methods research format in our study furthermore explicitly favoured the qualitative data over the quantitative outcome classification as focus of investigation, limiting the quantitative strand to a single, although psychometrically sound and often used, outcome measure. Our selection of patients based on self-reported symptoms nevertheless, had a considerable impact on our findings. With use of other means for categorisation, the sample likely would have turned out differently (e.g., using a different measure, multiple measures or relying on patients' satisfaction). Yet, as the use of statistical classification of clinically meaningful outcome (cf. Jacobson and Truax, 1991) is increasingly common in RCTs and standard outcome research at large (De Los Reyes et al., 2011), this study explicitly aimed to relate the exploration of patients' experiences to the much-used classification tool. Therefore, the aim of the current study was not to address the issue of measurement as such, nor the validation of the specific questionnaire that was used, but to deepen the understanding of outcome that is gained by these much used categories. Our conclusions on the relation between quantitative and qualitative appraisals of outcome can however not be generalised to the entire field of quantitative outcome evaluation that undoubtedly has evolved in the past decades, for instance with an increasing focus on person-centered questionnaires (Elliott et al., 2016). For the purpose of our study, an explanatory sequential design was most suited (Hesse-Biber, 2010). Nevertheless, further research aiming at different approaches to mixing methods and including idiosyncratic quantitative outcome evaluation could contribute greatly to our knowledge on outcome and psychotherapy.

Given the controlled context of our study (as data was collected in the context of a broader RCT), it offers a strong level of control for confounds. For instance, the research sample was characterised by a primary disorder of major depression, outcome was systematically evaluated in all patients and treatments were manualised. A potential threat is therefore, however, the external validity of the findings (Westen et al., 2004). Unlike in naturalistic studies, patients with more complex and acute psychopathologies were excluded. Nonetheless, all patients in our study showed comorbid disorders in line with clinical reality; for instance, the co-occurrence of major depression and anxiety disorders observed in this study is a robust finding throughout patient groups (cf. Hirschfeld, 2001). The participants in this study resembled a homogenous and local (predominately Caucasian, Flemish) group of patients, however. Specific (e.g., cultural, ethnic) or more diverse groups of patients could be the focus of complementing research. The research findings might also be biased by a selection of patients willing to participate in the study. Moreover, it is known that patients do not easily disclose negative experiences with therapy or with their therapist, and although interviews can enhance this openness (McLeod, 2000), in general, socially desirable answers cannot be excluded (Thurin and Thurin, 2007).

The model of nonimproved outcome must be considered tentatively, and we do not wish to make strong causal claims regarding the effectiveness of treatment or the causal influence of the therapist. In agreement with Strupp and Hadley (1977), we emphasise that the patient perspective is only one perspective on outcome (e.g., in addition to therapist or societal perspectives), and therefore highlights certain elements while neglecting others. This limits the findings of this study, as previous research has shown patient, therapist, and observers' perspectives on outcome not always converge and all add valuable insights for clinical practice (Altimir et al., 2010). Nevertheless, integrating in-depth inquiry of patients' narratives in the form of mixed methods research is of considerable value to outcome research and the study of non-improvement. In general, we argue that further research investigating the complex phenomena of outcome and therapy effects should aim at an integration of multiple methods as well as perspectives to grasp the wider picture (McLeod, 2011).

## Conclusion

Non-improvement in psychotherapy from the perspective of depressed patients can be understood as being stuck between knowing versus doing, resulting in a stalemate. Patients described both positive changes on the level of insight, mental stability and personal strength. The remaining issues were characterised by an ambition to change but feeling an inability to do so. No change in depression symptoms based on standard pre-post outcome evaluation thus becomes a partial change when considering patients' experience and shows a more complex picture in line with the complexity of experiencing depression. Investigating non-improvement by integrating in-depth analyses of patients' narratives in the form of mixed methods research proves to be of considerable value for understanding (negative) outcome and treatment effects more general.

## Author Contributions

MDS: main author of the manuscript, contribution to data collection, main investigator responsible for data-analysis and interpretation; RM: conception and development of study design, coordinating contribution to data collection, contribution to data-analysis and interpretation, main reviewer of the manuscript; KV: contribution to data collection, contribution to data-analysis and -interpretation, reviewer of the manuscript; FT: contribution to data collection and reviewer of the manuscript revision; MD: conception and development of study design, coordinating contribution to data collection, reviewer of the manuscript.

### Conflict of Interest Statement

The authors declare that the research was conducted in the absence of any commercial or financial relationships that could be construed as a potential conflict of interest.

## Footnotes

^1^Note of nuance: as outcome is predominantly evaluated by means of symptom-based scales (Braakmann, 2015), throughout this paper we will refer to a decrease in symptoms, even so we acknowledge outcome measurement is not limited to symptom-based scales but can also contain measures of general wellbeing, satisfaction and interpersonal functioning.

^2^Given the focus of the current study on patients being treated for major depression, the BDI-II-NL was selected as the outcome measure in this study. A complete overview of all measures used in the GSP can be found in the study protocol (Meganck et al., 2017).

^3^See McLeod (2013) for a summary of qualitative studies on this topic, Bohart and Wade (2013) on the role of agency, Castonguay and Hill (2007) for an elaboration on the role of insight in psychotherapy.

## References

[B1] Altimir C. Krause M. de la Parra G. Dagnino P. Tomicic A. Valdés N.. (2010). Clients', therapists', and observers' agreement on the amount, temporal location, and content of psychotherapeutic change and its relation to outcome. Psychother. Res. 20, 472–487. 10.1080/10503301003705871 20552535

[B2] APA (2013). American Psychiatric Association. Diagnostic and Statistical Manual of Mental Disorders, 5th Edn. Arlington, VA: American Psychiatric Association Publishing.

[B3] Baldwin A. S. Berkeljon A. Atkins D. C. Joseph O. A. Nielsen L. S. (2009). Rates of change in naturalistic psychotherapy: contrasting dose-effect and good-enough level models of change. J. Consult. Clin. Psychol. 77, 203–211. 10.1037/a0015235 19309180

[B4] Barkham M. Connell J. Stiles W. B. Miles J. N. V. Margison F. Evans C.. (2006). Dose-effect relations and responsive regulation of treatment duration: the good enough level. J. Consult. Clin. Psychol. 74, 160–167. 10.1037/0022-006X.74.1.160 16551153

[B5] Barlow D. H. (2010). Negative effects from psychological treatments: a perspective. Am. Psychol. 65, 13–20. 10.1037/a0015643 20063906

[B6] Barrett M. S. Chua W.-J. Crits-Cristoph P. Gibbons M. B. Casiano D. Thompson D. (2008). Early withdrawal from mental health treatment: implications for psychotherapy practice. Psychotherapy 45, 247–267. 10.1037/0033-3204.45.2.247 19838318 PMC2762228

[B7] Beck A. T. Steer R. A. Brown G. K. (1996). Manual for the Beck Depression Inventory II. San Antonio, TX: Psychological Corporation.

[B8] Beutler L. E. Moleiro C. Talebi H. (2002). Resistance in psychotherapy: what conclusions are supported by research. J. Clin. Psychol. 58, 207–217. 10.1002/jclp.1144 11793333

[B9] Binder P. E. Holgersen H. Nielsen G. H. (2009). What is a “good outcome” in psychotherapy? a qualitative exploration of former patients' point of view. Psychother. Res. 20, 258–294. 10.1080/10503300903376338 19941195

[B10] Blanton H. Jaccard J. (2006). Arbitrary metrics in psychology. Am. Psychol. 61, 27–41. 10.1037/0003-066X.61.1.27 16435974

[B11] Bockting C. Huibers M. (2011). Protocollaire behandeling van patiënten met een depressieve stoornis [protocol treatment for patients with depressive disorder], in Protocollaire Behandelingen Voor Volwassenen Met Psychiatrische Klachten [Protocol treatments for adults with psychiatric complaints], Vol. 1, eds Keijsers G. van Minnen A. Hoogduin K. (Amsterdam: Boom).

[B12] Bohart A. (1993). Experiencing: the basis of psychotherapy. J. Psychother. Integr. 3, 51–67. 10.1037/h0101189

[B13] Bohart C. A. Wade A. M. (2013). The client in psychotherapy, in Bergin and Garfield's Handbook of Psychotherapy and Behaviour Change, 6th Edn. ed Lambert M. J. (Hoboken, NJ: John Wiley and Sons, Inc), 219–257.

[B14] Bordin E. (1979). The generalizability of the psychoanalytic concept of the working alliance. Psychotherapy 16, 252–260. 10.1037/h0085885

[B15] Boswell J. F. Kraus D. R. Miller S. D. Lambert M. J. (2015). Implementing routine outcome monitoring in clinical practice: benefits, challenges, and solutions. Psychother. Res. 25:619. 10.1080/10503307.2013.817696 23885809

[B16] Bowie C. McLeod J. McLeod J. (2016). ‘It was almost like the opposite of what i needed': a qualitative exploration of client experiences of unhelpful therapy. Couns. Psychother. Res. 16, 79–87. 10.1002/capr.12066

[B17] Boyatzis R. E. (1998). Transforming Qualitative Information: Thematic Analysis and Code Development. London: Sage.

[B18] Braakmann D. (2015). Historical paths in psychotherapy research, in Psychotherapy Research: Foundations, Process and Outcome, eds Gelo O. C. G. Pritz A. Rieken B. (Vienna: Springer), 39–66. 10.1007/978-3-7091-1382-0_3

[B19] Castonguay L. Barkham M. Lutz W. McAleavey A. (2013). Practice-oriented research: approaches and applications, in Bergin and Garfield's Handbook of Psychotherapy and Behaviour Change, 6th Edn. ed Lambert M. J. (Hoboken, NJ: John Wiley and Sons, Inc), 85–133.

[B20] Castonguay L. G. Hill C. E. (2007). Insight in Psychotherapy. Washington, DC: American Psychological Association.

[B21] Charmaz K. (2014). Constructing Grounded Theory, 2nd Edn. Thousand Oaks, CA: Sage.

[B22] Cooper M. (2008). Essential Research Findings in Counselling and Psychotherapy: The Facts are Friendly. Los Angeles, CA: Sage.

[B23] Creswell J. W. Miller D. L. (2000). Determining validity in qualitative inquiry. Theory Into Pract. 39, 124–130. 10.1207/s15430421tip3903_2

[B24] De Los Reyes A. Kundey S. M. A. Wang M. (2011). The end of the primary outcome measure: a research agenda for constructing its replacement. Clin. Psychol. Rev. 31, 829–838. 10.1016/j.cpr.2011.03.011 21545781

[B25] De Smet M. Meganck R. (2018). Understanding long-term outcome from the patients' perspective: a mixed methods naturalistic study on inpatient psychotherapy. Psychol. Belg. 58, 1–21. 10.5334/pb.432 30479822 PMC6196577

[B26] Denzin N. K. Lincoln Y. S. (2005). The Sage Handbook of Qualitative Research. Thousand Oaks, CA: Sage.

[B27] Drisko J. (2004). Common factors in psychotherapy outcome: meta-analytic findings and their implications for practice and research. Fam. Soc. 85, 81-90. 10.1606/1044-3894.239

[B28] Elliott R. Slatick E. Urman M. (2001). Qualitative change process research on psychotherapy: alternative strategies, in Qualitative Psychotherapy Research: Methods and Methodology, eds Frommer J. Rennie D. L. (Lengerich: Pabst Science), 69–111.

[B29] Elliott R. Wagner J. Sales C. M. D. Rodgers B. Alves P. Cafe M. J. (2016). Psychometrics of the personal questionnaire: a client-generated outcome measure. Psychol. Assess. 28, 263–278. 10.1037/pas0000174 26075406

[B30] Faragher E. B. Cass M. Cooper C. L. (2005). The relationship between job satisfaction and health: a meta-analysis. Occup. Environ. Med. 62, 105–112. 10.1136/oem.2002.006734 15657192 PMC1740950

[B31] Fassinger R. E. (2005). Paradigms, praxis, problems, and promise: grounded theory in counseling psychology research. J. Couns. Psychol. 52, 156–166. 10.1037/0022-0167.52.2.156

[B32] First M. B. Spitzer R. L. Gibbon M. Williams J. B. W. (2002). Structured Clinical Interview for DSM-IV-TR Axis I Disorders, Research Version, Patient Edition. (SCID-I/P). New York,NY: Biometrics Research, New York State Psychiatric Institute.

[B33] Giorgi A. Giorgi B. M. (2003). The descriptive phenomenological psychological method, in Qualitative Research in Psychology: Expending Perspectives in Methods and Design, eds Camic P. M. Rhodes J. E. Yardley L. (Washington, DC: American Psychological Association), 243–274. 10.1037/10595-013

[B34] Glaser B. G. Strauss A. L. (1967). The Discovery of Grounded Theory: Strategies for Qualitative Research. Hoboken, NJ: Transaction publishers.

[B35] Hamilton M. (1967). Development of a rating scale for primary depressive illness. Br. J Soc. Clin. Psychol. 1967, 278–96. 10.1111/j.2044-8260.1967.tb00530.x 6080235

[B36] Hatcher R. L. Barends A. W. (2006). How a return to theory could help alliance research. Psychotherapy 43, 292–299. 10.1037/0033-3204.43.3.292 22122100

[B37] Hesse-Biber S. N. (2010). Mixed Methods Research, Merging Theory with Practice. New York, NY: Guilford press.

[B38] Hill C. (2004). Helping Skills: Facilitating Exploration, Insight, and Action, 2nd Edn. Washington, DC: American Psychological Association.

[B39] Hill C. E. Chui H. Baumann E. (2013). Revisiting and reenvisioning the outcome problem in psychotherapy: an argument to include individualized and qualitative measurement. Psychotherapy 50, 68–76 10.1037/a0030571 23505982

[B40] Hill C. E. Thompson B. J. Williams E. N. (1997). A guide to conducting consensual qualitative research. Couns. Psychol. 25, 517–572. 10.1177/0011000097254001

[B41] Hirschfeld R. M. A. (2001). The Comorbidity of major depression and anxiety disorders: recognition and management in primary care. Prim. Care Companion J. Clin. Psychiatry 3, 244–254. 10.4088/PCC.v03n0609 15014592 PMC181193

[B42] Jacobson N. S. Roberts L. J. Berns S. B. McGlinchey J. B. (1999). Methods for defining and determining the clinical significance of treatment effects: description, application, and alternatives. J. Consult. Clin. Psychol. 67, 300–307. 10.1037/0022-006X.67.3.300 10369050

[B43] Jacobson N. S. Truax P. (1991). Clinical significance: a statistical approach to defining meaningful change in psychotherapy research. J. Consult. Clin. Psychol. 59, 12–19. 10.1037/0022-006X.59.1.12 2002127

[B44] Jennissen S. Huber S. Ehrenthal J. C. Schauenburg H. Dinger U. (2018). Association between insight and outcome of psychotherapy: systematic review and meta-analysis. Am. J. Psychiatry. 175, 961–969. 10.1176/appi.ajp.2018.17080847 30068262

[B45] Kazdin A. E. (2001). Almost clinically significant (p<.10): current measures may only approach clinical significance. Clin. Psychol 8, 455–462. 10.1093/clipsy.8.4.455

[B46] Kazdin A. E. (2006). Arbitrary metrics: implications for identifying evidence-based treatments. Am. Psychol. 61, 42–71. 10.1037/0003-066X.61.1.42 16435975

[B47] Kazdin A. E. (2008). Evidence-based treatment and practice - new opportunities to bridge clinical research and practice, enhance the knowledge base, and improve patient care. Am. Psychol. 63, 146–159. 10.1037/0003-066X.63.3.146 18377105

[B48] Kazdin A. E. (2011). Single-Case Research Designs: Methods for Clinical and Applied Settings, 2nd Edn. Oxford: Oxford University Press.

[B49] Klein M. J. Elliott R. (2006). Client accounts of personal change in process experiential psychotherapy: a methodologically pluralistic approach. Psychother. Res. 16, 91–105. 10.1080/10503300500090993

[B50] Lambert M. (1992). Implications of outcome research for psychotherapy integration, in Handbook of Psychotherapy Integration, eds Norcross J. Goldstein J. (New York, NY: Basic Books), 94–129.

[B51] Lambert M. J. (2007). Presidential address: what we have learned from a decade of research aimed at improving psychotherapy outcome in routine care. Psychother. Res. 17, 1–14. 10.1080/10503300601032506

[B52] Lambert M. J. (2011). What have we learned about treatment failure in empirically supported treatments? some suggestions for practice. Cogn. Behav. Pract. 18, 413–420. 10.1016/j.cbpra.2011.02.002

[B53] Lambert M. J. (2013). The efficacy and effectiveness of psychotherapy, in Bergin and Garfield's Handbook of Psychotherapy and Behaviour Change, 6th Edn. ed Lambert M. J. (Hoboken, NJ: John Wiley and Sons, Inc), 169–218.

[B54] Lambert M. J. Barley D. E. (2001). Research summary on the therapeutic relationship and psychotherapy outcome. Psychotherapy 38, 357–361. 10.1037/0033-3204.38.4.357

[B55] Lambert M. J. Hansen N. B. Bauer S. (2008). Assessing the clinical significance of outcome results, in Evidence-Based Outcome Research. A Practical Guide to Conducting Randomized Controlled Trials for Psychosocial Interventions, eds Nezu A. M. Nezu C. M. (New York, NY: Oxford University Press, Inc.), 359–378.

[B56] Lambert M. J. Ogles B. M. (2009). Using clinical significance in psychotherapy outcome research: the need for a common procedure and validity data. Psychother. Res. 19, 493–501. 10.1080/10503300902849483 20183403

[B57] Lampropoulos G. K. (2011). Failure in psychotherapy: an introduction. J. Clin. Psychol. 67, 1093–1095. 10.1002/jclp.20858 21964920

[B58] Leichsenring F. Schauenburg H. (2014). Empirically supported methods of short-term psychodynamic therapy in depression – towards an evidence-based unified protocol. J. Affect. Disord. 169, 128–143 10.1016/j.jad.2014.08.007 25194781

[B59] Levitt H. M. Pomerville A. Surace F. I. (2016). A qualitative meta-analysis examining clients' experiences of psychotherapy: a new agenda. Psychol. Bull. 142, 801–830. 10.1037/bul0000057 27123862

[B60] Lilliengren P. Werbart A. (2005). A model of therapeutic action grounded in the patients' view of curative and hindering factors in psychoanalytic psychotherapy. Psychotherapy 42, 324–339. 10.1037/0033-3204.42.3.324

[B61] Luborsky L. (1984). Principles of Psychoanalytic Psychotherapy. A Manual for Supportive Expressive Treatment. New York, NY: Basic Books.

[B62] Mash E. J. Hunsley J. (1993). Assessment considerations in the identification of failing psychotherapy: bringing the negatives out of the darkroom. Psychol. Assess. 5, 292–301. 10.1037/1040-3590.5.3.292

[B63] Mays D. T. Franks C. M. (1985). Negative Outcome in Psychotherapy and What to do About it. New York, NY: Springer.

[B64] McElvaney J. Timulak L. (2013). Clients' experience of therapy and its outcomes in ‘good' and ‘poor' outcome psychological therapy in a primary care setting: an exploratory study. Couns. Psychother. Res. 13, 246–253. 10.1080/14733145.2012.761258

[B65] McLeod J. (2000). The contribution of qualitative research to evidence-based counselling and psychotherapy, in Evidence-Based Counselling and Psychological Therapies: Research and Applications, eds Rowland N. Goss S. (London: Routledge), 111–126.

[B66] McLeod J. (2011). Qualitative Research in Counselling and Psychotherapy. London: Sage.

[B67] McLeod J. (2013). Qualitative research. Methods and contributions, in Bergin and Garfield's Handbook of Psychotherapy and Behavior Change, 6th Edn. Lambert M. J. (New York, NY: John Wiley and Sons), 49–84.

[B68] Meganck R. Desmet M. Bockting C. Inslegers R. Truijens F. De Smet M.. (2017). The Ghent Psychotherapy Study (GPS) on the differential efficacy of supportive-expressive and cognitive behavioral interventions in dependent and self-critical depressive patients: study protocol for a randomized controlled trial. Trials 18, 1–11. 10.1186/s13063-017-1867-x 28292331 PMC5351275

[B69] Midgley N. Ansaldo F. Target M. (2014). The meaningful assessment of therapy outcomes: incorporating a qualitative study into a randomized controlled trial evaluating the treatment of adolescent depression. Psychotherapy 51, 128–137. 10.1037/a0034179 24377403

[B70] Mohr D. C. (1995). Negative outcome in psychotherapy: a critical review. Clin. Psychol. 2, 1–27. 10.1111/j.1468-2850.1995.tb00022.x

[B71] Mortelmans D. (2011). Kwalitatieve Analyse Met Nvivo.[Qualitative Analyses With Nvivo]. Leuven: Acco.

[B72] Mortelmans D. (2013). Handboek kwalitatieve onderzoeksmethoden, 4th Edn. [Handbook of Qualitative Research Methods]. Leuven: Acco.

[B73] Nezu A. M. Ronan G. F. Meadows E. A. McClure K. S. (2000). Practioner's Guide to Empirically Based Measures of Depressions. Dordrecht: Kluwer Academic Publishers.

[B74] Nilsson T. Svensson M. Sandell R. Clinton D. (2007). Patients' experiences of change in cognitive-behavioural therapy and psychodynamic therapy: a qualitative comparative study. Psychother. Res. 20, 37–41. 10.1080/10503300601139988

[B75] Norcross J. C. Wampold B. E. (2011). What works for whom: tailoring psychotherapy to the person. J. Clin. Psychol. 67, 127–132. 10.1002/jclp.20764 21108312

[B76] Ogles B. M. Lunnen K. M. Bonesteel K. (2001). Clinical significance: history, application, and current practice. Clin. Psychol. Rev. 21, 421–446. 10.1016/S0272-7358(99)00058-6 11288608

[B77] Paulson B. L. Everall R. D. Stuart J. (2001). Client perceptions of hindering experiences in counselling. Couns. Psychother. Res. 1, 53–61. 10.1080/14733140112331385258

[B78] Ratcliffe M. (2014). Experiences of Depression: A Study in Phenomenology. Oxford: OUP.

[B79] Stiles W. B. (1993). Quality control in qualitative research. Clin. Psychol. Rev. 13, 593–618. 10.1016/0272-7358(93)90048-Q

[B80] Stiles W. B. (2015). Theory-building, enriching, and fact-gathering: alternative purposes of psychotherapy research, in Psychotherapy Research: General Issues, Process, and Outcome, eds Gelo O. Pritz A. Rieken B. (New York,NY: Springer-Verlag), 159–179. 10.1007/978-3-7091-1382-0_8

[B81] Strauss A. Corbin J. (1990). Basics of Qualitative Research Techniques and Procedures for Developing Grounded Theory. London: Sage Publications.

[B82] Strupp H. H. Hadley S. W. (1977). A tripartite model of mental health and therapeutic outcome: with specific reference to negative effects in psychotherapy. Am. Psychol. 32, 187–196. 10.1037/0003-066X.32.3.187 848783

[B83] Svanborg C. Bäärnhielm S. Åberg Wistedt A. Lützen K. (2008). Helpful and hindering factors for remission in dysthymia and panic disorder at 9-year follow-up: A mixed methods study. BMC Psychiatry 8:52. 10.1186/1471-244X-8-52 18590579 PMC2481244

[B84] Thurin J.-M. Thurin M. (2007). Évaluer les psychothérapies, méthodes et pratiques. Paris: Dunod.

[B85] Timulak L. Creaner M. (2010). Qualitative meta-analysis of outcome of person-centred/experiential psychotherapies, in Person-Centred and Experiential Psychotherapies Work, eds Cooper M. Watson J. Hollendampf D. (Ross-on-Wye: PCCS Books), 66.

[B86] Timulak L. Lietaer G. (2001). Moments of empowerment: a qualitative analysis of positively experienced episodes in brief person-centered counselling. Couns. Psychother. Res. 1, 62–73. 10.1080/14733140112331385268

[B87] van der Does A. J. W. (2002). BDI-II-NL. Handleiding. De Nederlandse versie van de Beck Depression Inventory, 2nd Edn. Lisse: Harcourt Test Publishers.

[B88] von Below C. Werbart A. (2012). Dissatisfied psychotherapy patients: a tentative conceptual model grounded in the participants' view. Psychoanal. Psychother. 26, 211–229. 10.1080/02668734.2012.709536

[B89] von Below C. Werbart A. Rehnberg S. (2010). Experiences of overcoming depression in young adults in psychoanalytic psychotherapy. Eur. J. Psychother. Couns. 12, 129–147. 10.1080/13642537.2010.482745

[B90] Wampold B. E. (2007). Psychotherapy: the humanistic (and effective) treatment. Am. Psychol. 62, 855–873. 10.1037/0003-066X.62.8.857 18020771

[B91] Wampold B. E. Imel Z. E. (2015). The Great Psychotherapy Debate: The Evidence for What Makes Psychotherapy Work. New York, NY: Routledge 10.4324/9780203582015

[B92] Werbart A. von Below C. Brun J. Gunnarsdottir H. (2015). Spinning one's wheels”: nonimproved patients view their psychotherapy. Psychother. Res. 25, 546–564. 10.1080/10503307.2014.989291 25517205

[B93] Westen D. Novotny C. M. Thompson-Brenner H. (2004). The empirical status of empirically supported psychotherapies: assumptions, findings, and reporting in controlled clinical trials. Psychol. Bull. 130, 631–663. 10.1037/0033-2909.130.4.631 15250817

[B94] WHO (2017). World Health Organization. Depression Fact Sheet. Available online at: http://www.who.int/mediacentre/factsheets/fs369/en/

[B95] Wierzbicki M. Pekarik G. (1993). A meta-analysis of psychotherapy dropout. Prof. Psychol. 24, 190–195. 10.1037/0735-7028.24.2.190

[B96] Zimmerman M. Martinez J. A. Attiullah N. Friedman M. Toba C. Boerescu D. A. (2012). Why do some depressed outpatients who are in remission according to the Hamilton depression rating scale not consider themselves to be in remission? J. Clin. Psychiatry 73, 790–795. 10.4088/JCP.11m07203 22569085

[B97] Zimmerman M. McGlinchey J. B. Posternak M. A. Friedman M. Attiullah N. Boerescu D. (2006). How should remission from depression be defined? the depressed patient's perspective. Am. J. Psychiatry 163, 148–150. 10.1176/appi.ajp.163.1.148 16390903

